# Seventeenth Annual Meeting of the Illinois State Medical Society, Springfield, June 4th and 5th, 1867

**Published:** 1867-07

**Authors:** 


### SEVENTEENTH ANNUAL MEETING OF THE ILLINOIS STATE MEDICAL SOCIETY—SPRINGFIELD, JUNE 4TH AND 5TH, 1867.

---

The members of the Illinois State Medical Society assembled in Annual Session, in the Representatives' Hall, June 4th, 1867, at 10 o'clock A.M.

Dr. F. B. Haller, of Vandalia, President, called the Society to order, and introduced the Rev. Albert Hale, who opened the exercises with prayer.

Dr. N. Wright, Chairman of the Committee of Arrangements, then extended a cordial and appropriate welcome to the members of the Society; after which, the Assistant-Secretary, Dr. P. H. Bailhache, reported the following list of members and delegates as present:—

Drs. L. T. Hewins and T. N. Booe, of Loda, and D. L. Jewett, of Watseka, Iroquois Co.

Dr. C. Goodbrake, Clinton, DeWitt Co.

Drs. H. H. Roman, H. C. Barrell, P. J. Wardner, Justus Townsend, B. F. Stephenson, A. Trapp, Lyman B. Slater, H. B. Buck, Geo. T. Allen, B. M. Griffith, P. H. Bailhache, and H. K. Palmer, of Springfield, and N. Wright, of Chatham, Sangamon Co.

Dr. G. W. Albin, Neoga, Cumberland Co.

Drs. A. Niles, Joseph Robbins, Louis Watson, and J. T. Wilson, of Quincy, Adams Co.

Dr. F. H. VanEaton, Carrollton, Greene Co.

" M. F. DeWitt, Whitehall, "

" F. B. Haller, Vandalia, Fayette Co.

Drs. E. W. Moore, S. T. Trowbridge, J. A. W. Hostetler, and A. McBride, of Decatur, and H. N. Clark, of Niantic, Macon Co.

Dr. M. Reese, Abingdon, Knox Co.

Drs. D. Prince, G. R. Bibb, and W. S. Edgar, of Jacksonville, and John W. Craig, of Arcadia, Morgan Co.

Dr. J. T. Frazer, Howard's Point, Fayette Co.

Drs. D. W. Young and E. H. Gale, of Aurora, Kane Co.

Dr. D. S. Jenks, Plano, Kendall Co.

Drs. W. A. Knox, A. Fisher, N. S. Davis, H. A. Johnson, E. Powell, J. H. Hollister, J. Adams Allen, Moses Gunn, J. S. Hildreth, E. L. Holmes, R. N. Isham, F. O. Earle, DeLaskie Miller, C. T. Fenn, E. Ingals, and Frank W. Rielly, Chicago, Cook Co.

Dr. T. D. Washburn, Hillsboro, Montgomery Co.

Drs. E. P. Cook and J. C. Corbus, Mendota, LaSalle Co.

Dr. J. F. Potts, Peoria, Peoria Co.

" J. Little, Leroy, McLean Co.

" H. Noble, Heyworth, McLean Co.

Drs. S. W. Noble, D. O. Crist, D. L, Crist, and T. F. Worrell, Bloomington, McLean Co.

Dr. S. B. McGlumphy, Lincoln, Logan Co.

" J. S. Whitmire, Metamora, Woodford Co.

" O. Q. Herrick, Kansas, Edgar Co.

" H. W. Davis, Paris, Edgar Co.

Drs. J. O. Hamilton, J. L. White, and G. C. Lyon, Jerseyville, Jersey Co.

Dr. Wm. Massie, Grandview, Edgar Co.

" W. R. Fox, Wilmington, Will Co.

Drs. W. H. Veatch and John Ewing, Pawnee, Sangamon Co.

Dr. H. S. Hurd, Galesburg, Knox Co.

" B. K. Shurtleff, Amboy, Lee Co.

